# Synthesis, Spectroscopic, and Antimicrobial Studies on Bivalent Zinc and Mercury Complexes of 2-Formylpyridine Thiosemicarbazone

**DOI:** 10.1155/2009/851316

**Published:** 2009-04-29

**Authors:** Sulekh Chandra, Shikha Parmar, Yatendra Kumar

**Affiliations:** ^1^Department of Chemistry, Zakir Husain College, University of Delhi, Jawaharlal Nehru Marg, New Delhi 110002, India; ^2^ITS Paramedical College (Pharmacy), Delhi Meerut Road, Muradnagar, Ghaziabad 201206, India

## Abstract

A series of metal complexes of Zn(II) and Hg(II) having the general composition [M(L)_2_]X_2_ [where L = 2-formylpyridine thiosemicarbazone; M = Zn(II) and Hg(II); X = Cl^−^, NO_3_^−^ and 1/2SO_4_^2−^] have been prepared and characterized by elemental chemical analysis, molar conductance, and spectral (IR and mass) studies. The IR spectral data suggests the involvement of sulphur and azomethane nitrogen in coordination to the central metal ion. On the basis of spectral studies, a tetrahedral geometry has been assigned for Zn(II) and Hg(II) complexes. The free ligand and its metal complexes have been tested in vitro against a number of microorganisms in order to assess their antimicrobial properties.

## 1. Introduction

Thiosemicarbazones are very versatile ligands. They can coordinate to metals as neutral molecules or, after deprotonation, as anionic ligands, and can adopt a variety of different coordination modes [1]. The mono-thiosemicarbazones usually behave as tridentate ligands while the bis-thiosemicarbazones normally bind to the metal center through the two S atoms, the two azomethine nitrogen, and the pyridine nitrogen [2]. Interest in metal complexes with thiosemicarbazones and semicarbazone ligands has been stimulated because biological activities are often enhanced on complexation. Thiosemicarbazones and their metal complexes have received considerable attention because of their antibacterial, antifungal, antitumor, antiamoebic, antimalarial, antiviral, radioprotective, trypanocidal, and anti-inflammatory activities [3–14].

The biological activity is considered to involve three kinds of mechanisms: (i) inhibition of enzyme ribonucleoside diphosphate reductase (essential for DNA synthesis); (ii) creation of lesions in DNA strand by oxidative rupture; (iii) binding to the nitrogen bases of DNA or RNA, hindering or blocking base replication [15].

**Figure 1:**
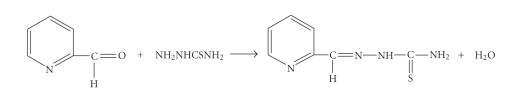
Synthesis and structure of ligand.

In view of the above applications, the present work relates to the synthesis, spectroscopic, and antimicrobial studies on Zn(II) and Hg(II) complexes of 2-formylpyridine thiosemicarbazone. The ligand used in the study is depicted in Figure 1.

## 2. Experimental

### 2.1. Materials

All the chemicals used were of Anala R grade and procured from Sigma-Aldrich and Fluka. Metal salts were purchased from E. Merck and used as received.

### 2.2. Synthesis of Ligand (L)

Hot ethanolic solution of thiosemicarbazide (4.55 g, 0.05 mol) and 2-formylpyridine (4.75 mL, 0.05 mol) were mixed slowly with constant stirring. This mixture was refluxed at 70–80°C for 2 hours. On cooling, a cream colored compound was precipitated out. It was filtered, washed with cold EtOH, and dried under vacuum over P_4_O_10_ (yield (65%), mp 210°C). Elemental chemical analysis data is shown in Table 1.

### 2.3. Synthesis of Complexes

Hot ethanolic solution (20 mL) of ligand (0.02 mol) and hot ethanolic solution (20 mL) of the corresponding metal salts (0.01 mol) were mixed together with constant stirring. The mixture was refluxed for 3-4 hours at 70–80°C. On cooling, colored complexes were precipitated out. They were filtered, washed with 50% ethanol, and dried under vacuum over P_4_O_10_.

### 2.4. Analysis

The C, H, and N were analyzed on Carlo-Erba 1106 elemental analyzer. The nitrogen content of the complexes was determined using Kjeldahl's method. Zinc and mercury metal ions were determined complexometrically. Molar conductance was measured on the ELICO (CM82T) conductivity bridge. Electronic impact mass spectrum was recorded on JEOL, JMS-DX-303 mass spectrometer. IR spectra (KBr) were recorded on FTIR spectrum BX-II spectrophotometer. The molecular weights of the complexes were determined cryoscopically in benzene.

### 2.5. Antimicrobial Screening

In vitro antimicrobial screening was performed by the agar disc diffusion method [16, 17]. All the test organisms were obtained from Microbial Type Culture Collection and Gene Bank (MTCC), Institute of Microbial Technology, Chandigarh, India. Nutrient agar growth media was prepared according to the instructions of MTCC. 25 mL nutrient agar media was poured in each petriplate of 90 mm diameter. The inoculum was spread on the top of solidified media. Sterile discs of Whatmann no. 1 filter paper having a diameter of 6 mm, impregnated with the test compounds, were placed at four equidistant places on the inoculated petriplates. The zone of inhibition was calculated in millimeters.

#### 2.5.1. Antibacterial Screening

The antibacterial activity of the ligand and its metal complexes were tested against gram-positive (*Staphylococcus aureus and Staphylococcus epidermides*) and gram-negative (*Escherichia coli and Pseudomonas aeruginosa*) pathogenic bacteria at a concentration of 100 *μ*gdisc^−1^. Nutrient agar media was prepared by using peptone, beef extract, yeast extract, NaCl, agar-agar, and distilled water. Bacterial cultures were adjusted to 0.5 McFarland turbidity standard and inoculated onto the nutrient agar plates [18]. The discs were carefully transferred onto the seeded agar plates. Filter paper disc treated with DMSO served as control and, Amikacin (30 *μ*gdisc^−1^) was used as a standard drug. All determinations were made in duplicate for each of the compounds. An average of two independent readings for each compound was recorded. The petriplates were incubated at 37°C for 24 hours. The zone of inhibition was calculated.

#### 2.5.2. Antifungal Screening

The antifungal activity of the ligand and its metal complexes were tested against two pathogenic fungi, *Candida albicans* and *Aspergillus niger* at a concentration of 200 *μ*gdisc^−1^ for each. Nystatin was used as standard fungicide, and DMSO served as a means of control. For *Candida albicans,* nutrient agar media was prepared using yeast extract, peptone, dextrose, agar-agar, and distilled water. Inoculum suspension in normal saline was prepared from fresh, mature (3 to 5 days old) cultures grown on nutrient agar slants. Using spectrophotometry at 530 nm, turbidity was measured and adjusted to match a 0.5 McFarland density standard resulting in an inoculum containing 1 × 10^6^ to 5 × 10^6^ fungal cells/mL [19]. This suspension was used to directly inoculate agar plates.

For *Aspergillus niger*, nutrient agar media was prepared using czapek concentrate (NaNO_3_, KCl, MgSO_4_ · 7H_2_O, FeSO_4_ · 7H_2_O, and distilled water), K_2_HPO_4_, yeast extract, sucrose, agar-agar, and distilled water. Seven days old colonies were covered with approximately 1 mL of sterile 0.85% saline, and the suspensions were made by gently probing the colonies. The resulting mixture of conidia and hyphal fragments was withdrawn and transferred to sterile tube. After heavy particles were allowed to settle for 3 to 5 minutes, the upper homogenous suspensions were collected. The densities of the conidial suspensions were read and adjusted to an optical density (OD) that ranged from 0.09 to 0.11 (80% to 82% transmittance) at 530 nm [20]. The sterile discs impregnated with the test compounds were placed on the already seeded plates at 30°C for 48 hours. A clearing zone around the disc indicated the inhibition activity of the test compounds on the pathogenic fungi.

## 3. Results and Discussion

**Figure 2:**
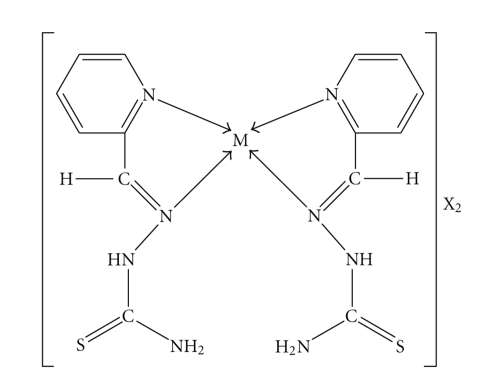
Suggested structure of complex, where M = Zn(II) and Hg(II) and X = Cl^−^, NO_3_^−^ and 1/2SO_4_^2−^.

The complexes were synthesized by reacting ligand with the metal ions in 2 : 1 molar ratio in an ethanolic medium. The ligand that behaves as bidentate coordinates through the N_azomethane_ and N_pyridine_ chelating centers (Figure 2). Elemental analysis of complexes corresponds to the composition as shown in Table 1. All the complexes are found to be soluble in DMSO and DMF, sparingly soluble in water and ethanol, and insoluble in acetone. The molar conductance measurements of the complexes in DMF lies in the range of 122–140 Ω^−1^cm^2^mol^−1^, indicating their 1 : 2 electrolytic behavior. Thus, the complexes may be formulated as [M(L)_2_]X_2_, (where M = Zn(II) and Hg(II); L = 2-formylpyridine thiosemicarbazone; X = Cl^−^, NO_3_^−^ and 1/2SO_4_^2−^).

## 4. Mass Spectrum

**Figure 3:**
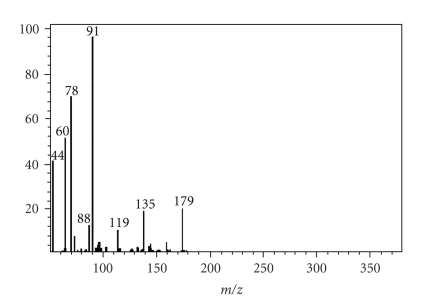
Electronic impact mass spectra of ligand (L).

The electronic impact mass spectrum of the ligand (Figure 3) shows the final peak at 179 amu [(C_7_H_8_N_4_S), calculated atomic mass 180 amu], and other peaks like 44, 60, 78, 88, 91, 119, and 135 amu may correspond to various fragments. The weak peak described at 135 amu is assigned to the fragment [C_6_H_8_N_4_]^+^, corresponding to the loss of CS group. A very weak peak at 119 amu is assigned to the fragment [C_6_H_6_N_3_]^+^, corresponding to the loss of CSNH_2_ group. The most intense peak at 91 corresponds to the fragment [C_6_H_5_N]^+^. Other peaks at 88, 78, 60, and 44 correspond to the fragments [CH_3_N_3_S]^+^, [C_5_H_4_N]^+^, [CSNH_2_]^+^, and [CS]^+^, respectively.

**Figure 4:**
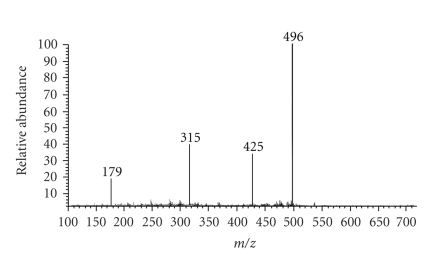
Electronic impact mass spectra of [Zn(L)_2_]Cl_2_.

Complex [Zn(L)_2_]Cl_2_ shows a single peak at 496 amu, which coincides with that of molecular ion. Loss of two chloride ions is in agreement with a peak at 425 amu. Loss of one of the ligands is in agreement with a peak at 315 amu. A single peak at 179 amu coincides with that of 2-formylpyridine thiosemicarbazone (Figure 4). Complex [Zn(L)_2_](NO_3_)_2_ shows one peak at 551 amu, which coincides with that of molecular ion. Loss of one of the ligands is in agreement with a peak at 370 amu. [Zn(L)_2_](SO_4_), [Hg(L)_2_]Cl_2_, [Hg(L)_2_](NO_3_)_2_, and [Hg(L)_2_](SO_4_) show peaks at 520, 633, 687, and 655 amu, respectively, which are in agreement with their molecular formulae.

## 5. Infrared Spectrum

The assignments of the significant IR spectral bands of ligand and its metal complexes are presented in Table 2. The highest frequency band of the 2-formylpyridine thiosemicarbazone at 3429 cm^−1^ can be assigned to asymmetric *ν*(N–H) vibration of the terminal NH_2_ group. The other bands at 3267 and 3164 cm^−1^ may be due to the symmetric *ν*(N–H) vibrations of the imino and amino groups. A band at 1611 cm^−1^ in the IR spectra of the ligand is due to *ν*(C=N)_azomethane_ group. Coordination of azomethine nitrogen in complexes is suggested by the shift of *ν*(C=N)_azomethane_ band to lower frequencies along with the occurrence of *ν*(N=N) band at higher frequency in the IR spectra of complexes compared to the ligand. Coordination of imine nitrogen is also consistent with the presence of a band at 453–486 cm^−1^, assignable to *ν*(M–N). Another band at 557 cm^−1^ in the free ligand is due to *ν*(C=N)_pyridine_ group and is also shifted toward higher frequency. This indicates that the nitrogen atom of the pyridine group is also involved in complex formation. The thioamide *ν*(C=S) band at 776 cm^−1^ of free ligand is not shifted on complexation which indicates the noninvolvement of sulfur in coordination [21]. The absence of large systemic shift of *ν*_as_(NH_2_) and *ν*_sym_(NH_2_) modes to lower frequencies indicates no interaction between the terminal amino nitrogen and the metal ions. In each complex, two 2-formylpyridine thiosemicarbazone ligands coordinate to the central metal ion through two pyridine N atoms and two azomethine N atoms. Thus, it is concluded that the ligand acts as a bidentate chelating agent.

## 6. Anions

The infrared spectra of the nitrate complexes show sharp and strong band at 1384 cm^−1^, characteristic for uncoordinated nitrate group [22]. IR bands in the region of 1408–1426 and 615–622 cm^−1^, characteristic of uncoordinated sulfate group, are seen in the infrared spectra of sulfate complexes [23].

## 7. Antimicrobial Studies

**Zinc Complexes**

Results of bactericidal screening show that the free ligand (L) was much more active than its zinc complexes, while the antifungal results show that all the zinc complexes are more active than the free ligand. The variation in the effectiveness of different compounds against different organisms depends either on the impermeability of the cells of the microbes or the difference in ribosomes of microbial cells [24]

**Mercury(II) Complexes**

The antimicrobial screening data shows that the ligand exhibits antimicrobial properties, and it is important to note that the Hg(II) metal chelates exhibit more inhibitory effect than the parent ligand. From Table 3, it is clear that the zone of inhibition is much larger for metal chelates against gram-positive (*Staphylococcus aureus and Staphylococcus epidermides*) and gram-negative (*Escherichia coli and Pseudomonas aeruginosa*) pathogenic bacteria. The increased activity of metal chelates can be explained on the basis of chelation theory. It is known that the chelation tends to make the ligand act as a more powerful and potent bactericidal agent, thus killing more of the bacteria than the ligand. It is observed that, in a complex, the positive charge of the metal is partially shared with the donor atoms present in the ligand, and there may be *π*-electron delocalization over the whole chelating [25]. This increases the lipophilic character of the metal chelate and favors its permeation through the lipoid layer of the bacterial membranes. There are also other factors which increase the activity, namely solubility, conductivity, and bond length between the metal and the ligand.

The result of fungicidal screening (Table 4) shows that Hg(II) complexes were more active than the free ligand against pathogenic fungi, *Candida albicans* and *Aspergillus niger *. The mode of action may involve the formation of a hydrogen bond through the azomethane nitrogen atom with the active centers of the cell constituents, resulting in interference with the normal cell process [24].

## Figures and Tables

**Table 1:** Analytical data for the ligand and its Zn(II) and Hg(II) complexes.

Compounds	Molecular formulae	Atomic mass found (calcd)	Yield (%) (g)	Color	Mp (°C)	Analysis found (calcd)				Molar conductance (Ω^−1^cm^2^mol^−1^)
						C	H	N	M	
Ligand (L)	C_7_H_8_N_4_S	179(180)	65	Shiny	210	46.67	4.45	31.11	—	—
			(4.97)	Cream		(46.62)	(4.49)	(31.20)	—	
[Zn(L)_2_]Cl_2_	ZnC_14_H_16_N_8_S_2_Cl_2_	496(495)	61	Off white	285	33.95	3.26	22.59	13.15	122
			(3.01)			(33.93)	(3.23)	(22.62)	(13.13)	
[Zn(L)_2_](NO_3_)_2_	ZnC_14_H_16_N_10_S_2_O_6_	551(549)	59	Milky	212	30.58	2.96	20.43	11.85	128
			(3.23)	Yellow		(30.60)	(2.91)	(20.40)	(11.83)	
[Zn(L)_2_](SO_4_)	ZnC_14_H_16_N_8_S_3_O_4_	520(521)	55	Yellow	>350	32.29	3.09	21.43	12.44	125
			(2.86)			(32.24)	(3.07)	(21.49)	(12.47)	
[Hg(L)_2_]Cl_2_	HgC_14_H_16_N_8_S_2_Cl_2_	633(631)	62	Off white	225	26.67	2.57	17.78	31.89	133
			(3.91)			(26.62)	(2.53)	(17.74)	(31.85)	
[Hg(L)_2_](NO_3_)_2_	HgC_14_H_16_N_10_S_2_O_6_	687(685)	60	Dark	125	24.58	2.36	20.40	29.31	140
			(4.11)	brown		(24.52)	(2.33)	(20.43)	(29.34)	
[Hg(L)_2_](SO_4_)	HgC_14_H_16_N_8_S_3_O_4_	655(657)	60	Brown	180	25.53	2.40	17.09	30.55	138
			(3.94)			(25.57)	(2.43)	(17.04)	(30.59)	

**Table 2:** Important infrared spectral bands (cm^−1^) and their assignments, where s = strong; ms = medium strong; m = medium; mw = medium weak; w = weak.

Compounds	Assignments				
	*ν*(N–H)	*ν*(N=N)	*ν*(C=N)_azomethane_	*ν*(C=N)_pyridine_	*ν*(M–N)
C_7_H_8_N_4_S ligand (L)	3267 s	1107 s	1611 s	557 m	—
[Zn(L)_2_]Cl_2_	3275 s	1183 s	1589 s	664 m	474 m
[Zn(L)_2_](NO_3_)_2_	3275 m	1177 s	1588 m	661 m	478 m
[Zn(L)_2_](SO_4_)	3286 m	1167 ms	1560 m	673 m	486 mw
[Hg(L)_2_]Cl_2_	3292 ms	1155 m	1594 s	626 m	460 w
[Hg(L)_2_](NO_3_)_2_	3295 s	1171 m	1551 m	621 m	459 m
[Hg(L)_2_](SO_4_)	3273 s	1163 m	1599 ms	621 m	453 w

**Table 3:** Antibacterial screening data of the ligand and its Zn(II) and Hg(II) complexes.

Compounds	(Conc.) *μ*g/disc	Diameter of zone of inhibition (mm)			
		Gram positive		Gram negative	
		*Staphylococcus aureus*	*Staphylococcus epidermidis*	*Escherichia coli*	*Pseudomonas aeruginosa*
Ligand (C_7_H_8_N_4_S)	100	20	16	18	08
[Zn(L)_2_]Cl_2_	100	10	09	09	10
[Zn(L)_2_](NO_3_)_2_	100	12	10	08	08
[Zn(L)_2_]SO_4_	100	08	09	07	07
[Hg(L)_2_]Cl_2_	100	24	20	22	16
[Hg(L)_2_](NO_3_)_2_	100	25	18	21	12
[Hg(L)_2_]SO_4_	100	20	17	19	10
Amikacin	30	26	22	21	20

**Table 4:** Antifungal screening data of the ligand and its Zn(II) and Hg(II) complexes.

Compounds	Conc. (*μ*g/disc)	Diameter of zone of inhibition (mm)	
		*Candida albicans*	*Aspergillus niger *
Ligand (C_7_H_8_N_4_S)	200	14	—
[Zn(L)_2_]Cl_2_	200	15	14
[Zn(L)_2_](NO_3_)_2_	200	16	12
[Zn(L)_2_](SO_4_)	200	14	07
[Hg(L)_2_]Cl_2_	200	20	22
[Hg(L)_2_](NO_3_)_2_	200	16	16
[Hg(L)_2_](SO_4_)	200	12	08
Nystatin	200	26	18
